# An Outline of the Allen Method

**Published:** 1919-05-24

**Authors:** 


### TREATMENT OF DIABETES.

### An Outline of the Allen Method.

Experience has shown that Dr. Allen's treatment in Diabetes, which was published three years ago, constitutes a distinct advance on previous methods in that in suitable cases it brings about a rapid disappearance of glycosuria and affords an opportunity of keeping the urine free from sugar for long periods on an adequate diet.

### Essentials of the Method.

Essentially the procedure is as follows:—The patient is allowed water, tea, coffee, and meat extracts only, with possibly a small quantity of cream, say ½ oz. per diem, and occasionally whisky or brandy at night, until his urine has been free from sugar for twenty-four hours. Food is then given, beginning on the first day with green vegetables, say 4 oz., and butter ½ oz., and two eggs. The next day the allowance of vegetables is increased and a small quantity of meat may be tried, say 3 oz. On the third or fourth day, provided the urine is still free from sugar, the addition of starchy foods is begun, so that usually five or six grm. of starch are added every other day, protein, or protein and fat, being added on the intervening day until the allowance of these is sufficient. A simple way to add the carbohydrate is to allow 1 oz. potato on the fourth and fifth days, 2 oz. potato on the sixth and seventh, and omit the potato, substituting 1 oz. bread, on the eighth and ninth days, and so on. When sugar returns, it is usually sufficient to omit the starchy foods completely for one day and return rapidly to an allowance of starch distinctly less than that at which sugar returned. This allowance may then be very slowly increased.

As the diet increases more variety can be introduced. Besides regulating the carbohydrate, the total energy value of the diet has to be considered. Allen found that in experimental diabetes the animals had less glycosuria and survived longer when they were underfed, than when they were fed freely without carbohydrate, and the same applies to man. A good rule is to insist on the weight being kept distinctly below normal. An allowance of 2,000 calories a day with, say 70 grm. carbohydrate is found to be sufficient for a moderately active life, though if hard work is undertaken more will be necessary. Even during the fast it is not necessary to keep the patient in bed, and for the remainder of the treatment a certain amount of exercise is desirable, as the tolerance both for food in general and for carbohydrate is increased thereby.

Frequently the patient is instructed how to test his own urine for sugar and is provided with a list from which he can vary his diet without altering his intake. The vegetables and the caloric value of the diet need not be rigidly regulated provided they conform generally to prescription; but for starchy foods and fruits strict limitation is necessary, according to the tolerance established.

### Control Information.

The information required is as follows:—

*Vegetables* with 5 per cent. carbohydrates or less, 7 calories per ounce, greens, cauliflower, marrow, onions, green beans, rhubarb, tomatoes, celery, etc.

*Vegetables and Fruit*, 10 per cent. carbohydrate or less, 10 calories per ounce, turnips, swedes, carrots, beetroot, oranges, strawberries, unripe fruits.

*Carbohydrate* 15 *per cent.*, 15 calories per ounce, parsnips, artichokes, apples, bananas, many fruits; plums and grapes have 20 per cent.

*Important foods, carbohydrate per cent., calories per ounce*, oatmeal 60 per cent., 110; flour 75 per cent., 100; bread 50 per cent., 75; milk 5 per cent., 20; cream 4 per cent., 70; butter nil, 225; cheese 2 per cent., 110; meat nil, 80; fish (cod) nil, 15; eggs nil, 70 calories each; nuts 5 per cent., 75.

Cases who respond to treatment can avoid glycosuria with reasonable care for indefinite periods, though it would be an exaggeration to describe them as cured; and sudden reappearances of the sugar occasionally occur, which are met by a temporary diminution of the carbohydrate allowance. Obviously, for success it is important to deal with a case in the early stages of the disease; but in this connection it is well to note that among slight glycosurias there is a condition called *diabetes innocens*, in which the sugar in the blood is not above normal and the urinary sugar is small in amount and does not tend to increase. These cases, in which Allen's treatment is neither necessary nor desirable, can be distinguished by determination of the sugar in the blood, and by observation of the urine.

The most satisfactory cases for treatment are young adults, and it is just these who would be expected to be rapidly progressive without treatment. In senior diabetics the response is less marked; but where the treatment is practicable it has a distinct beneficial influence on diabetic gangrene and on sepsis.

### Failures Explained.

The treatment thus outlined may fail in two ways:—(1) Glycosuria may disappear with fasting, but reappear persistently before an adequate diet is reached. In some of these it may be possible to avoid glycosuria by the use of a diet containing little or no carbohydrate beyond that of the vegetables of the first group, giving an occasional fast of one day when sugar returns. If this is not practicable the case can be classed with the second group. (2) Glycosuria diminishes but does not disappear with starvation, which for practical purposes should be limited to three or at most four days. In these

**Treatment of Diabetes**—(*continued*).

cases the diet may be regulated and a periodical fast prescribed, say once a week; but it is debatable whether any real benefit is obtained by the repeated fast, except with reference to acidosis, as the patient is liable to get very weak and is perhaps more prone to infection.

SEVERE CASES.

IN the most severe cases, whose urine shows a well-marked reaction for diacetic acid, the influence of fasting is beneficial in that the diacetic acid diminishes both during the fast and in the period of feeding immediately following the fast, but a similar result is also obtained by limiting the diet to vegetables, oatmeal, and a little milk, thus combining the influence of partial starvation with that of a considerable intake of carbohydrate, both of which are known to diminish excessive acidosis. The latter procedure is probably preferable in the worst cases.

The influence of fasting on the acidosis of diabetics is the opposite of that in normal individuals, in whom fasting causes diacetic acid to appear in the urine in small quantity. Where there is no previous acidosis, the diabetic behaves in this respect in the same way as the normal; just as with the normal, the intensity of the diacetic acid reaction obtained varies greatly with different individuals, and there appear to be some who starve badly in this respect, giving an excessively marked reaction in the urine by the second day with possibly other symptoms of acidosis. In such a case, it is necessary to give carbohydrate food at once. Previous preparation with a diet poor in fat is said to be useful, but instances of this complication are rare and can easily be dealt with when they occur if the urine is regularly tested during the fast.

